# Upconversion Luminescence Sensitized pH-Nanoprobes

**DOI:** 10.3390/molecules22122064

**Published:** 2017-11-25

**Authors:** Manoj Kumar Mahata, Hyeongyu Bae, Kang Taek Lee

**Affiliations:** Department of Chemistry, School of Physics and Chemistry, Gwangju Institute of Science and Technology, Gwangju 61005, Korea; mkmahata@gist.ac.kr (M.K.M.); hgbae3609@gist.ac.kr (H.B.)

**Keywords:** optical sensors, molecular probes, pH-sensors, upconversion, photoluminescence

## Abstract

Photon upconversion materials, featuring excellent photophysical properties, are promising for bio-medical research due to their low autofluorescence, non-cytotoxicity, low photobleaching and high photostability. Upconversion based pH-nanoprobes are attracting considerable interest due to their superiority over pH-sensitive molecular indicators and metal nanoparticles. Herein, we review the advances in upconversion based pH-nanoprobes, the first time in the seven years since their discovery in 2009. With a brief discussion on the upconversion materials and upconversion processes, the progress in this field has been overviewed, along with the toxicity and biodistribution of upconversion materials for intracellular application. We strongly believe that this survey will encourage the further pursuit of intense research for designing molecular pH-sensors.

## 1. Introduction

Optical imaging and sensing are considered significant tools for the characterization of biological samples. In the recent years, a number of optical probing systems have been developed, which are suitable in biological environment, for example, fluorescent probes could be used to locate the position of protein and monitor biological processes [1,2]. Quantum dots capable of being employed in biological sensing and imaging are some other probes used for in vivo applications [3,4]. However, the probes fail in the case of deeper penetration depth in the samples. Some remaining drawbacks of common fluorescent probes include low intensity, high photobleaching, light scattering and significant autofluorescence from the background. Real-time monitoring of pH and temperature in living cells is necessary to know the cellular mechanisms. Concurrently, gases such as oxygen, carbon-dioxide and ammonia are involved in a number of bio-chemical reactions and their concentrations need to be measured in clinical research and diagnostics [5]. Thus, there is a constant interest for novel nanomaterials with improved features for biomedical applications.

Thus far, a number of fluorescent systems have been developed for pH-sensing [6,7,8,9]. The ratiometric method of pH-sensors is based on the two fluorescence emissions with their main advantage of minimum dependence on the measuring environment and conditions. Usually, different pH-sensitive dyes are loaded with reference dyes or nanoparticles, such as quantum dots (CdSe/ZnSe/ZnS), silica (core-cell), latex polymer dots, etc. [10,11,12]. However, they have strong side effects due to their excitation with ultraviolet (UV) or visible light and dyes have poor photostability, which limits their versatility. These problems can be overcome by realizing photon upconversion materials, which are used in pH-sensing and are favorable to be applied in complex biological environments [13,14].

Rare earth (RE) doped upconverting nanoparticles (UCNP) have a unique capability to convert low energy infrared (IR) photons into visible photon in a successive absorption process. These nanoparticles impart many advantages and overcome most of the drawbacks of fluorescent dyes and quantum dots [15,16]. UCNPs have been employed in various fields of applications, for instance, solar cell concentrator, three-dimensional volumetric display, gas sensing, temperature sensing, bio-molecule sensing, etc. [17,18,19,20,21]. Upconversion (UC) emission is obtained from the RE incorporated inorganic host materials, which are superior in terms of deep tissue penetration, low photo-damage of biological cell and less cytotoxicity that makes them perfect for biophysical application. Remarkably, we have observed stochastic photon emission in microsecond and nanosecond scale in UCNPs in our latest research [22]. The UC emission is achieved with low-cost, low-power, readily available lasers and has advantages over simultaneous two-photon absorption and second harmonic generation, which require high-cost, high-intense laser sources. Five UC processes, namely, excited state absorption (ESA), co-operative upconversion (CU), energy transfer upconversion (ETU), energy migration upconversion (EMU) and photon avalanche (PA), are briefly illustrated in Section 3. Most UCNPs rely on 980 nm excitation to take advantage of Yb^3+^ ions, which have very high absorption cross-section at that wavelength [23,24]. Water has very high absorption at 974 nm wavelength. However, the local minima of water absorption are at the wavelength of 800 nm which is regarded as ideal excitation wavelength with less heating to the tissues. The water absorption at 800 nm is 20 times less than that at 974 nm. Nd^3+^ ions are usually used to absorb 800 nm light. For detailed information on synthesis and other applications (except pH-sensing) of UC materials, the reader is directed to previous reviews [25,26,27,28].

UCNPs can be excited by wavelength 700–1000 nm, where the absorption coefficients of water, hemoglobin and other biomatter are minimum [29]. For example, a self-ratiometric pH-nanoprobe using NaYF_4_:Tm^3+^/Yb^3+^ and fluorescein (FITC) has been developed, in which the UC emissions upon 980 nm laser light excitation, in the blue (475 nm) and red (645 nm) regions, are realized as response and reference signals, respectively, for ratiometric pH-determination [30]. A molecular pH-probe bound to the surface of UCNPs (coated with PEI) has been used to obtain resonance energy transfer from UCNPs to the pHrodo^TM^ Red dye, which absorbs the green (550 nm) emission band of NaYF_4_:Er^3+^/Yb^3+^ as the source of excitation [31]. Most of those are highlighted on the surface modified UC nanoparticles with a pH-indicator. Only a few of these have no pH-sensitive dyes, rather they utilize the quenching and recovery properties of UCNPs when nanohybrids of UCNPs are prepared with quenchers [32,33]. Nevertheless, the thin films of pH-nanoprobes are required to design for their practical applications in flowing liquids. In a recent attempt, a pH-sensor designed as a proof-of-concept on the interaction of bromocresol green-doped silica and UCNPs has been devised to detect pH in implanted medical devices, caused by bacteria [34]. Moreover, the response, reversibility and testing of the pH-nanoprobes are important issues.

High precision, intra-cellular pH-quantification is one of the most important requirements in biomedical research. A considerable development in pH-sensitive drug delivery systems based on various surface ligands attached to the surface of nanoparticles have been noticed [35]. However, our knowledge is extremely limited on what happens after cell internalization to these nanoparticles. Some studies assume that the pH-sensitive drug delivery system terminates in the acidic compartment. However, it is not yet adequately tested for confirmation. Some earlier reports claim that surface modification of UCNPs has a vital role in cellular entry through the plasma membrane [36,37]. To develop photoluminescence methods for imaging and sensing of pH, several optical and chemical probes have been put forward in the last two decades. It is very important to know the pH of cytoplasm for successful therapy. Monitoring the pH value also helps to understand phagocytosis and endocytosis in cells. The pH-sensing at the sub-cellular level is of great significance to understand intra-cellular trafficking in molecular biology and medicinal research. Intra-cellular pH has important role in cell behavior [31]. Abnormal pH may show cellular dysfunction and diseases such as cancer, neurological disorder, etc. Each compartment of cell has distinct pH values and gives different operational condition for each metabolic route. It is necessary to control pH of the cellular compartments to survive the organism. Therefore, intra-cellular pH measurement has particular interest to understand the cell metabolism, vesicle trafficking, membrane dynamics, etc. Thus, pH determination is of paramount importance for better understanding of cellular processes [31]. 

In this review, we start with the working principle of upconversion based pH-sensors and then describe the constitution of upconversion nanoparticles and the upconversion luminescence processes. The development of photon upconversion based pH-sensors is then thoroughly reviewed. The toxicity and biodistribution of the UCNPs inside cell have been discussed for their real-time application. Some developments in luminescent molecular indicators have been reviewed by Shi et al. [38], Schäferling et al. [3] and Wencel et al. [39]. Thus, the scope of this review is restricted to the photon upconversion based optical pH-nanoprobes, since its first report in 2009 to date and will pave way a broader outlook towards realistic design of pH-sensors.

## 2. Principle of pH-Sensor

**Figure 1:**
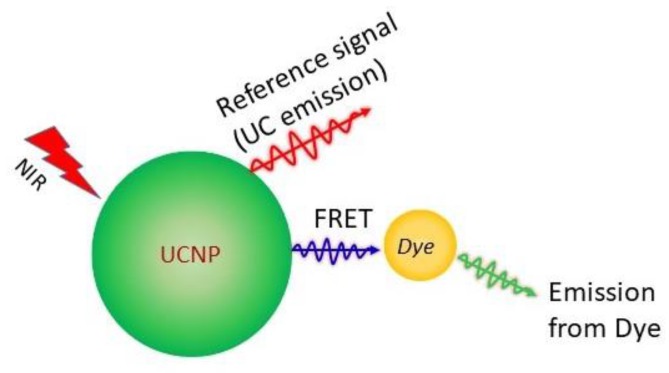
Schematic representation of upconversion sensitized pH-sensor. The pH-sensitive dye emission is analyzed with respect to the UC emission. The Förster resonance energy transfer (FRET) from UCNP to the pH-sensitive dye is shown.

RE-doped UCNPs, covering a long range of wavelength by UC emission are regarded as an excellent choice to facilitate reversible and referenced pH-sensing. In this context, several pH-sensors have been established by bonding UCNPs with pH-sensitive indicators as the pH-sensing probe. Among them, some have been developed as the pH-sensing membrane, while others are in nanoparticle or nanohybrid structure. The pH-sensitive dyes absorb at least one of the UC emissions and the pH is measured using the simple idea of the variation of absolute luminescence or ratiometric variation of intensity of emission bands. Förster resonant energy transfer (FRET) is an important process in evaluating the energy transfer between donor and acceptor [40]. The dipole-dipole interaction is generally strong. The solution of this interaction was derived by Förster and is known as FRET. The probability of FRET in a simple donor-acceptor pair depends on the radiative lifetime of the donor, and Förster distance (at which the FRET efficiency is 50%). The process is long range and the value of Förster distance is about 7 nm in organic molecules [41]. Recently, the modeling of energy migration through RE^3+^ ions show that the distance for energy migration can reach 10–30 nm [42,43]. The Förster resonant energy transfer (FRET) from UCNP to the pH-sensitive dye is considered to take place. A countable number of pH-sensors have been constituted using UCNPs and quenchers (such as graphene oxide, Ni-nanoparticles). They do not include pH-sensitive dyes and work on the principle of variation of absolute UC intensity with pH-change. The schematic representation of UC based pH-nanoprobes is shown in Figure 1.

In 1909, the pH of a solution was first defined by Søren Sørensen as the negative logarithm of hydronium ions (H_3_O^+^), though conventionally, it is called as proton or hydrogen ion [44,45].

(1) $$\mathrm{pH}=-\log{\left[H_{3}O\right]}^{+}f_{H^{+}}$$

where f_H_^+^ is activity coefficient and for ideal solution is close to unity. Equation (1) is applicable only in aqueous solution. According to Henderson-Hasselbalch equation, the pH is as measured

(2) $$\mathrm{pH}={\mathrm{pK}}_{a}+\log\frac{\left[A^{C-1}\right]}{\left[{\mathrm{HA}}^{C-1}\right]}$$

where pK_a_ is the negative logarithmic of the acidic dissociation constant K_a_. [A^c−1^] and [HA^c^] are the concentrations of basic and acidic forms of pH-sensitive dye, respectively. Equation (2) can only be used under the assumption that the initial concentration and equilibrium concentration are almost the same. This expression is useful for predicting pH in buffer. However, since the base and acid forms are based on initial values, it is not applicable for relatively strong acids or strong bases, and for solutions that are highly diluted or concentrated.

Equation (2) also gives the range of pH-determination of an indicator and in terms of activities, the pH-value is measured. The maximum pH-sensitivity is obtained close to the pK_a_ value. The pK_a_ value is dependent on the interaction between pH-indicator and its environment along with temperature [39,46]. Therefore, a fixed pH-value is desired for the pH-sensitive dyes for their application in intercellular medium. For high sensor resolution the response of signal should be notable within a very small change in pH and are crucial for intercellular and extracellular pH measurements, where the pH, in general, varies within 4.4 to 7.5. For instance, the extracellular pH is 7.35 to 7.45 and endosome, lysosome and cytosol have pH range within pH 4.4–6.0, 4.5–5.0 and 6.8–7.4, respectively. Thus, it is reasonable to design pH-probes with high sensitivity instead of wide range with low responsivity [3]. To calibrate pH inside living cell, a standard method for intercellular pH-calibration should be followed. The dual wavelength based optical fiber pH-sensors have high precision of pH (0.01 units) measurement, but similar precision cannot be attained inside the cells [3,47]. A further crucial concern is their pH-response. Some nanoprobes which have offered response time in their study are within 0.38 to 10 min in this review.

## 3. Structure of UCNPs and Upconversion Processes

The idea of photon upconversion was first put forward by Bloembergen while developing IR photon detectors in 1959 [48]. Later in 1960, F. Auzel observed IR to visible photon upconversion and explained the mechanism for Yb^3+^-doped Er^3+^ and Tm^3+^ systems. Further, in 2004, he studied the UC mechanism in detail [49]. However, in the early days of photon upconversion, the possible applications of these materials were not perceived because controlling the particle’s shape and size were not vastly possible that time. In the present century, since the tremendous progress in nanotechnology, the upconversion materials have been re-investigated. Most of the RE elements have similar electronic configuration ([Xe] 4f^n−1^5d^o−1^6s^2^), which allows for numerous intra-configurational transitions. The 4f electron in RE^3+^ ions (excluding scandium, yttrium, lanthanum and lutetium) are shielded by completely filled 5s^2^5p^6^ subshells which makes them less sensitive in the host lattice. Furthermore, they exhibit long lifetime, sharp emission lines and large anti-Stokes shift.

Luminescent upconversion nanoparticles are composed of inorganic host materials incorporated with a small amount of RE ions. Selection of REs is necessary to control the energy transfer among the REs, namely activator and sensitizer. The activators are compulsory luminescent centers in the inorganic host materials. For instance, the RE^3+^ ions such as Er^3+^, Tm^3+^, Ho^3+^, Eu^3+^, and Dy^3+^ are some commonly used activators which can be excited by monochromatic excitation sources. None of the host materials are efficient for achieving intense UC emission. They are different in respect of their co-ordination, energy transfer and dopant distribution [50,51]. Materials with low phonon energy are often chosen as host due to their low non-radiative rates, which does not reduce the emission efficiency. Although several upconversion materials show high intense emission, the selection of host, dopant and their concentration is important for designing efficient UCNPs. The series of fluoride host materials (NaXF_4_; X = Y, Gd, La) have very low phonon energy, making them effective host matrices for RE doping. Additionally, the chemical and thermal stability of these nanomaterials are a plus [52,53]. Among these series, the hexagonal structures of NaXF_4_ are more efficient than their cubic structures. The less-symmetrical crystal-field around REs in hexagonal form enhances the probability of Laporte forbidden intra-4f transitions. Some other excellent upconversion host matrices include phosphates (YPO_4_, GdPO_4_, LuPO_4_), vanadates (GdVO_4_, LaVO_4_, YVO_4_), oxides (ZrO_2_, Y_2_O_3_), etc., even though some of them have higher phonon energy [52]. The luminescence intensities are linked with the quantum-mechanical transition rates which are involved with the initial and final states of the transition and are approximately determined by Judd-Ofelt theory [54,55]. The theory approximates equal population of Stark manifolds and isotropic host matrix to estimate theoretically the radiative rates of the RE^3+^ ions. For an electric-dipole allowed transition, the radiative decay rate can be expressed as:

(3) $$W_{\mathrm{rad}}=\frac{4e^{2}\omega^{3}}{3\hbar c^{3}}\frac{1}{2J+1}n{\left(\frac{n^{2}+2}{3}\right)}^{2}\underset{\lambda \;=\;2,\;4,\;6}{\sum }\Omega_{\lambda }{\left|\langle \mathrm{SLJ}|\left|U^{\left(\lambda \right)}\right||S^{\prime }L^{\prime }J^{\prime }\rangle \right|}^{2}$$

where |SLJ〉 and |S′L′J′〉 are the initial and final states of the transition. e is electronic charge, ω is average angular frequency of the optical transition, ħ is reduced Planck constant, and c and n are the speed of light and refractive index respectively. The term [(n^2^ + 2)/3]^2^ is the Lorentz local field correction factor, and (2J + 1) is degeneracy of the initial state. |〈SLJ||U^(λ)^||S′L′J′〉|^2^ represents the squared reduced matrix elements of the unit tensor. The Ω_λ_ (λ = 2, 4, 6) are the Judd-Ofelt parameters which are obtained from the absorption spectra of the material and provide the effect of the host matrix on the transition probabilities (electric dipole). Precise understanding of the UC mechanisms requires to combine the magnetic dipole transitions to the Judd-Ofelt rates [54,55].

The environment of the dopant ions inside host lattice has a potential impact on the control of the luminescence properties. Low non-radiative decay rates, long-lived metastable states, high level of population of the excited states and evenly distribution of dopant ions are major requisite for the construction of efficient UCNPs. Quenching process reduces the quantitative amount of the excited states, giving rise to down-gradation of UC properties. Radiationless transitions are also associated with the presence of impurities and other nearby RE ions in the vicinity. The phonons play role in the bridging of ground and excited states, providing that the energy gap should be mediated by at most 5–6 phonons [56,57]. If the required numbers of phonons are more than that, the radiative processes become dominant. Hence, the materials with lower phonon frequency are a better choice. At the same time, the other properties of the host materials, such as chemical stability, should be considered. Host materials with Y^3+^, Na^+^, or Ca^2+^ as one of the elements are frequently used in UC systems because of their ideal ionic radii closer to the RE ions in their trivalent state, produce fewer lattice defects and lattice strains with little compromise for efficient UC emission [58,59,60].

Dopant ions serving as activator and sensitizer have a vital role as luminescent centers in the upconversion materials to yield high UC efficiency. The RE^3+^ ions possessing ladder like energy levels with long-lived energy states are exploited for doping in host materials.

Amongst the RE^3+^ ions, Er^3+^, Ho^3+^, and Tm^3+^ are used in most of the studies, as they are known to provide good emission intensity in the visible region of the electromagnetic spectrum. A list of RE ions in various host material [61,62,63,64,65,66,67,68,69] with their different UC emission bands upon 980 nm excitation is shown in Table 1. The energy levels of Er^3+^—^4^I_15/2_, ^4^I_13/2_, ^4^I_11/2_, ^4^F_9/2_ and ^2^H_11/2_/^4^S_3/2_ are suitable for 800 nm or 980 nm laser light excitation. Similarly, the other participating energy levels of Tm^3+^ are ^3^H_6_, ^3^F_4_, ^3^H_4_, ^1^G_4_, and ^1^D_2_, and of Ho^3+^ are ^5^I_8_, ^5^I_7_, ^5^I_6_, ^5^F_5_, ^5^F_4_, and ^5^S_2_. Other RE ions such as Dy^3+^, Pr^3+^, Tb^3+^, and Eu^3+^ have also played role as an activator in UC processes [70]. The proper concentration of activators is also a factor to control UC luminescence. There is an optimized concentration at which the emission intensity is maximum after that it decreases due to the increased energy transfer from one activator to another. Thus, sensitizers which transfer energy to the activator ions upon excitation, are introduced in the UC materials in order to enhance the UC process. Apart from this, most of the activated ions have small absorption cross-section at near-infrared (NIR) excitation wavelength, yielding low UC efficiency. The efficiency of the UC systems can be enhanced by many folds with the doping of sensitizer ions which have large absorption cross-section at the excitation wavelength. The Yb^3+^ is such a known and common sensitizer for Er^3+^, Tm^3+^, Eu^3+^, Ho^3+^, etc. activators due to its very high absorption cross-section at 980 nm wavelength. Due to the energy transfer from Yb^3+^ to other ions, it has been found that the emission intensity is increased by several folds for Er^3+^, Ho^3+^ and Tm^3+^ ions [71]. The upconversion nanomaterials sometimes suffer from weak luminescence and several methods have proven to enhance the emission intensity in an internal and external ways, for example, formation of core/shell nanoparticles, surface coating, varying crystal phases, doping with non-RE ions, etc. When some non-rare-earth ions are incorporated in the host materials, they break the crystal field symmetry around the RE ions. This results tailoring the upconversion properties; for instance, Zn^2+^, Na^+^, K^+^ have been used to enhance emission in La_2_O_3_, BaTiO_3_, CaMoO_4_, etc. matrices [72,73,74]. The surface of the UCNPs is coated with polycationic macromolecules, such a PEI, PAA, etc., which have been proven to enhance the performance of UCNPs in the surface-bound state [31]. Therefore, to boost the UC properties, the selection of dopants, their concentrations and surface coating should be considered carefully. 

**Figure 2:**
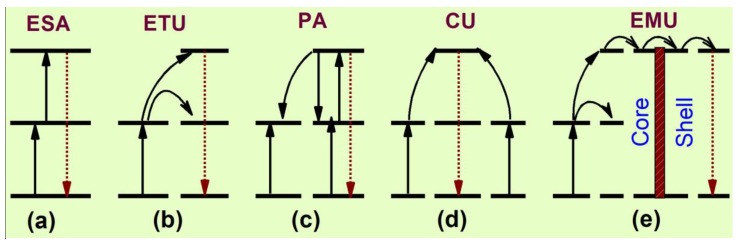
Schematic representation of different upconversion processes [47].

The basic UC mechanisms [52,75,76,77] involve five major processes, namely excited state absorption (ESA), energy transfer UC (ETU), photon avalanche (PA), cooperative UC (CU) and energy migration-mediated UC (EMU). The simplified UC processes have been depicted in Figure 2.

ESA is a sequential absorption of one or multiple photons from the ground state to the intermediate excited states, from which UC emission is obtained (Figure 2a). The excited ions are capable of absorbing second pump photon. This process is observed at higher dopant’s concentration of the activator ions and at higher concentration, it is likely to decrease the UC emissions intensity via radiationless relaxation. The efficiency of UC emission is suppressed because of the low absorption of RE ions, induced by parity forbidden transitions. The ETU process (Figure 2b) refers to energy transfer between two types of luminescent centers, namely sensitizer and activator. This process is more efficient than ESA. When the sensitizer is excited, the energy is transferred to the adjacent activator and luminescence occurs while the activator comes back to lower excited states or ground states. To have efficient energy transfer between sensitizer and activator, they should possess specific energy levels which are resonant with convenient spatial distance. Due to resonant energy transfer, the excitation time is longer that increases the probability of UC and makes it efficient [75,76]. The PA (Figure 2c) occurs beyond a critical value of pump density and is more complicated than the above two UC processes. When the pump density is very high, the intermediate levels of many RE ions become populated by ground state absorption and the population of the UC emitting level takes place through ESA or ETU from other excited ions. After that, sufficient cross-relaxation occurs among the ground state ions and excited state ions. This phenomenon increases the population of intermediate reservoir state and the UC emitting state. As a result, an “avalanche” of producing a large number of excited ions through feedback looping occurs and makes PA process the most efficient [77]. The CU (Figure 2d) process basically occurs in Yb^3+^/RE^3+^ doped UC materials. In CU, the UC emitting level of the activator ions is populated by two nearby sensitizer ions via co-operative energy transfer. The Yb^3+^ ions are known to play as sensitizer ions in Yb^3+^/RE^3+^ doped materials. The most recently discovered upconversion process is EMU (Figure 2e), which involves four types of ions namely sensitizer, accumulator, migrator and activator. The activator and sensitizer/accumulator ions located at different layers are linked by migrators. The sensitizer promotes the accumulator into the higher excited state via energy transfer and excited accumulator then migrates its energy to the migrators via the core-shell interface of the nanoparticle. The migrated energy is finally trapped in the shell by an activator, located at that layer and UC emission occurs. 

## 4. Development in Photon Upconversion Based pH-Probes

The NIR light falls within the biological transparency window (700–1300 nm) and its deep-penetration and low auto-fluorescence give low photostability in comparison to UV and visible light. Most of the optical pH-sensors are investigated to perform within this window [30,31,32,33,34,78,79,80,81,82,83,84,85,86,87,88]. A list of the UC materials speculating pH-sensing is given in Table 2.

**Figure 3:**
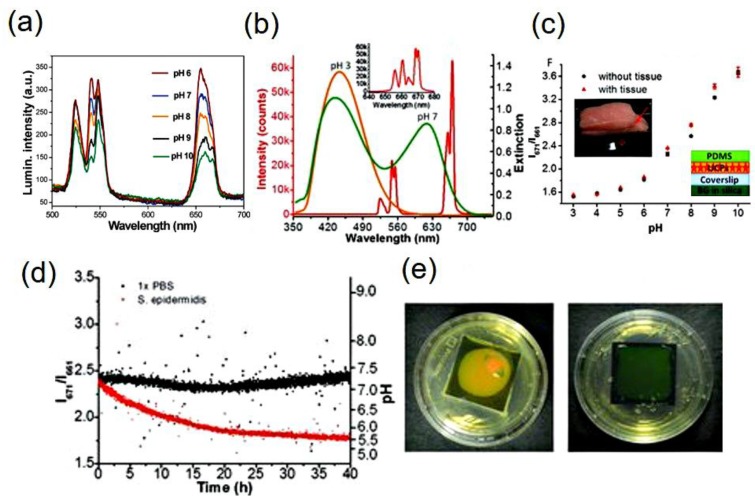
(**a**) pH-dependent upconversion emission spectra of bromothymal blue conjugated NaYF_4_:Er^3+^/Yb^3+^ film upon 980 nm excitation (Reproduced from Ref. [78] with permission from The Royal Society of Chemistry). (**b**) The upconversion spectrum of Y_2_O_2_S:Er_3+_/Yb_3+_ UCNPs (red line, left *y*-axis) and the extinction spectra (right *y*-axis) of bromocresol green-doped silica films in pH 3.0 (orange) and buffer 7.0 (green). Inset shows the red luminescence with higher resolution. (**c**) The calibration curves of bromocresol green conjugated Y_2_O_2_S:Er^3+^/Yb^3+^ films, without (black dots) and with (red points) passing through porcine tissue. Left inset shows the photograph of the sensor film sandwiched between two porcine tissues for pH calibration. The inset in the right side shows the schematic representation of the pH sensor. (**d**) pH determination in real-time with the sensor film passing through porcine tissue. The red dots exhibit the variation of pH due to the bacterial development at the interface of the TSA plate and pH-sensor; the black dots show the variation of pH of the controlled sample with same volume of phosphate buffer solution. The corresponding pH values, calculated according to the calibration curve, are shown in the right *y*-axis. (**e**) The photograph at the left side shows the pH sensor film by the end of the real-time experiment with *S. epidermidis* and at the right side shows the film of the control sample by the end of acquisition (Figure 3b–d: Reproduced from Ref. [34] with permission of John Wiley & Sons Ltd.).

The first approach to give an introduction of UC nanoparticles for pH-sensing is considered to be presented by Sun et al. in 2009 [78]. Since then, there has been a rapid rise in UC based pH-sensor. Nanorods of NaYF_4_:Er^3+^/Yb^3+^ with diameter ~50 nm and length ~950 nm were employed in the first study, as they give intense green (^2^H_11/2_/^4^S_3/2_→^4^I_15/2_) and red (^4^F_9/2_→^4^I_15/2_) emission from the Er^3+^ ions. Bromothymol blue, a non-toxic pH-sensitive indicator, used with UC nanorods to design the pH-sensors, have several advantages, such as non-self-fluorescent nature, obvious color change with pH and the suitable pK_a_ value ~6.82 (in water at 25 °C). The pH-indicator is likely to exert an inner filter effect on the UC emission, based on its basic or acidic form. Although the NaYF_4_:Er^3+^/Yb^3+^ nanorods are pH-insensitive, they show pH-dependent UC emission due to their energy transfer to the indicator (Figure 3a). In the history of UC based pH-sensing, the focus has always been given to the nanoparticles but those are not suitable for pH-sensing in flowing matter (e.g., blood and urine). Therefore, some studies were carried out with sub-micrometer films of pH-sensing nanomaterials.

### 4.1. Upconversion Based pH-Sensing Membranes

A further study [79] with Xylenol orange dye conjugated, silica coated UCNPs doped Tm^3+^ ions shows pH-dependent quenching and recovery of 450 nm emission (Tm^3+^:^1^D_2_→^3^F_4_). At low pH, the ratio of 450 nm emission to a reference emission (646 nm), decreases while at high pH, the ratio increases due to low absorption of the dye at the blue region, with a linear variation of the ratio in pH range 4–8. A sensor film of the same elements was designed of thickness ~12 µm. The membrane exhibits pH-sensitivity and significant change of UC emissions occurs at pH 10, as compared to pH 6.

UC based pH-sensor film could be used to detect bacterial infections in the implanted medical devices and a proof-of-concept was introduced by the group of Wang et al. [34]. Biofilm forms generally on the implanted devices and degrades the functions of the devices. Early detection and treatment of infections in these devices is difficult. Therefore, the development of pH-sensor film to detect bacterial growth in the implanted medical devices in a non-invasive way is promising in medical treatment. The sensor film is composed of two main layers: an UC luminescence layer (Y_2_O_2_S:Er^3+^/Yb^3+^) and a layer of pH-indicator (bromocresol green) [34]. The designed film is shown schematically in the inset (right side) of Figure 3c with the optical absorption and UC emission spectra of the pH-indicator and Y_2_O_2_S:Er^3+^/Yb^3+^ nanoparticles in Figure 3b. The luminescence emission bands are observed to change with pH. However, the luminescence from the dye is not possible to see because of its low emission intensity.

The red UC luminescence is composed of two emission bands arises from transitions from different Stark sub-levels of Er^3+^ ions and variation of these two bands peaking at 661 nm and 671 nm gives different intensity at different pH. Justification of the intensity variation is the protonation and deprotonation of the dye at various pH, as a clear isosbestic point in the absorption spectra is observed. The film response time for pH measurement has been tested as 10 minutes which is far below the time taken for acidification by bacteria. The performance test was carried out in porcine tissue with the designed pH-sensor film and it shows the variation of the intensity ratio of 671 nm over 661 nm as pH-dependent, without disturbing the shape of the UC spectra with reasonable response in the pH range 5–10. Finally, they pointed out that calibration curves with and without biological tissue do not exhibit any significant difference [34].

In real-time, the sensor was further tested to monitor the pH variation caused by bacteria on a tryptic soy agar (TSA) plate (Figure 3d). Interestingly, it was observed that the Staphylococcus epidermidis 35,984 bacteria makes colonies in thin film and changes its pH from 7.4 to 5.7 within 40 h. As a visible proof, the color of the film changes from green (neutral) to yellow (acidic) (Figure 3e). This study gives evidence that the UC based ratiometric pH-sensors could be potential for detection of bacterial infection on implanted devices in real-time [34]. 

**Figure 4:**
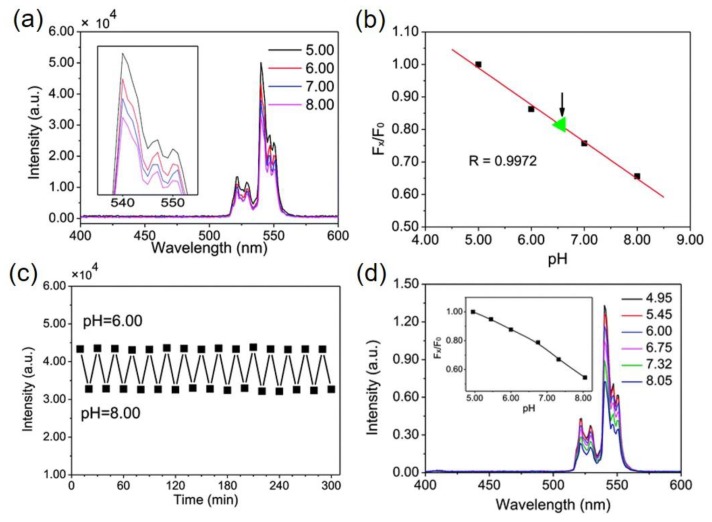
(**a**) The upconversion spectra of the graphene oxide–PEI-NaYF_4_; Er^3+^/Yb^3+^ film in buffer solutions upon excitation at 980 nm; (**b**) ratio of green emission intensity at pH 5 to green emission intensity at other pH values; (**c**) repetitive cycles of the pH-sensor between pH 6.00 and pH 8.00, showing response time; and (**d**) upconversion spectra of graphene oxide–PEI-NaYF_4_; Er^3+^/Yb^3+^ in urine of mice. Inset shows the ratio of green emission intensity at pH 5 to green emission intensity at other pH values plotted against the pH values in diluted urine (reproduced from Ref. [32] with permission from the PCCP Owner Societies).

Another idea came up by the group of Yan et al. [32] is to use nanohybrids for pH measurement. As graphene oxide is known to act as a quencher [32] for quantum dots and dyes, the same was utilized with the upconversion material as well. The energy transfer process in upconversion-graphene oxide nanohybrid is not inner filter effect, where graphene oxide acts as acceptor and upconversion material as donor. The linear change in luminescence intensity is noticed in Figure 4a,b within pH 5–8 using this nanostructure [32].

Thin film prepared using UCNPs and graphene oxide demonstrates that at pH 8 the green emission (540 nm) decreases to 35% in comparison to emission at pH 5. Next (Figure 4d) the film was used to measure the pH of urine of mice and shows 55% decrease in intensity at pH 8.5 compared to at pH 4.95, with response time less than 1 min (Figure 4c,d). The major cause lies between the interaction of negatively charged graphene oxide and positively charged polyethyleneimine functionalized UCNPs which is dependent on pH; the result is manifested in the upconversion luminescence upon excited by 980 nm light. This type of nanostructure, which does not require pH-sensitive dye to measure pH of a solution, has a new platform in the field of pH-sensing for their use as bio/chem sensing. In addition, due to low cost, non-toxicity and larger surface area, the graphene oxide is promising to prepare robust, free-standing hybrid films [32].

**Figure 5:**
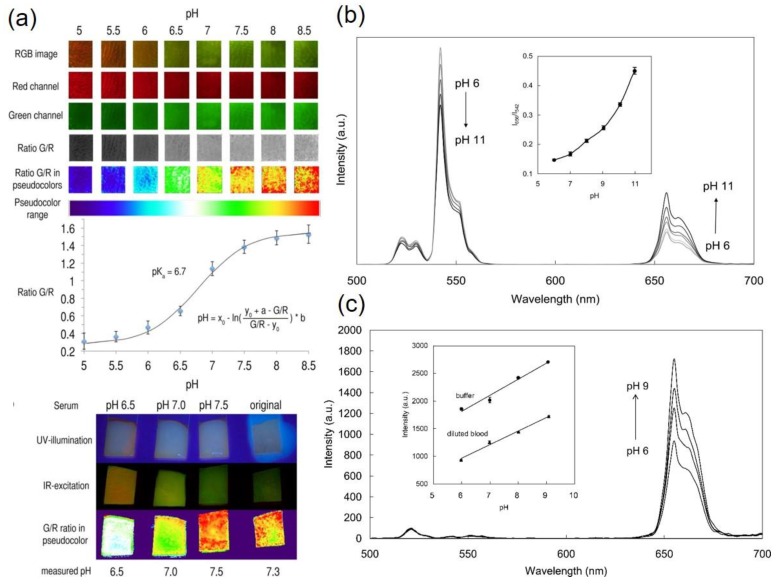
(**a**) Imaging calibration row (RGB) of the membrane. The graph at the middle of (**a**) shows the ratio G/R values versus the pH for calibration point. At the bottom, four sensor spots were impregnated with serum specimens. The first three samples have adjusted pH values at pH 6.5, pH 7.0, and pH 7.5 and the fourth one is original. Autofluorescence from background is seen under UV illumination (first row). IR excitation and RGB imaging of the real serum samples remains unaffected from the background (second row). The G/R ratio is shown in pseudocolor (bottom row) and converted into corresponding pH values (Adapted with permission from Ref. [76]. Copyright (2014) American Chemical Society.). (**b**) The 980 nm excited upconversion spectra of the pH-sensor film containing UCNPs (NaYF_4_; Er^3+^/Yb^3+^) and chromoionophore (ETH 5418), within pH values 6–11. Inset shows intensity ratio of 656 nm to 542 nm emission band as a function of pH; (**c**) Upconversion spectra of the pH-sensor film containing UCNPs and ETH 5418 in diluted blood. Inset shows the intensity variation of 656 nm emission band of sensor film in buffer and diluted blood as a function of pH (Figure 5b,c: Adapted with permission from Ref. [84]. Copyright (2012) American Chemical Society).

More recent evidence of an easy, inexpensive and straight design of a pH-sensor has been put forward recently by the group of Meier et al. [83]. The sensor film consists of upconversion phosphors and pH-sensitive dye hosted on a hydrogel matrix. The sensor film changes its color with pH-variation. The green UC emission at 550 nm is quenched and red UC luminescence at 660 nm is prominent in acidic medium (pH 5). Green emission becomes dominant as the pH increases (at pH 8.5). This enables to estimate the pH in naked eye. Another interesting feature of the sensor film is that it can sense and image of pH through ratiometric and referenced RGB technique by a digital color camera due to overlapping of UC emission of the sensor film with the red and green color channels of the digital camera. The detail of the methods of imaging technique is presented in Ref. [83]. Figure 5a shows the RGB-pH-imaging calibration and measurement of pH of human serum using the devised sensor film.

Explaining and justifying an optical sensor for pH-determination and metal ions sensing in whole blood and other biological specimens based on ion-exchange mechanism has drawn attention in a recent study [84]. The sensor film is composed of UCNPs (NaYF_4_; Er^3+^/Yb^3+^) and chromoionophore (ETH 5418), embedded in plasticized polyvinyl chloride matrix. The pH-indicator shows a clear isosbestic point at 577 nm. Its absorption maximum increases from green (525 nm) to red (680 nm) region due to deprotonation and protonation, respectively. The UC luminescence spectra of the sensor film upon 980 nm excitation at pH 6–11 shows that the green emission (^2^H_11/2_/^4^S_3/2_→^4^I_15/2_) decreases with increasing pH, while the red emission (^4^F_9/2_→^4^I_15/2_) increases with increasing pH (Figure 5b). The green and red UC emissions were decreased and increased by 27% and 56%, respectively at pH 11, as compared to those at pH 6, due to inner filter effect of the UCNPs and pH-indicator. Evaluation of the sensor film was performed in blood specimen and it was observed that the green UC emission is reduced by a large amount while the red UC was weakened by 50% and served to measure pH through intensity variation. The red UC emission increases linearly with the pH of blood as shown in Figure 5c, confirming that the designed membrane is capable of measuring pH at the complex environment of blood. Together with the pH measurement of blood, the sensor also revealed that it can detect metal ions, such as Na^+^ and Ca^2+^ at the same time [84].

### 4.2. Upconversion Nanoparticles as pH-Sensors

In a systematic study [85], aminosilane coated NaYF_4_; Er^3+^/Yb^3+^ have been covalently coupled with pHrodoTM Red indicator to obtain energy transfer from UCNPs to the indicator directly. In a long range of pH from acidic (pH 4) to basic (pH 8), the nanoprobe exhibits its pH-sensitivity. The luminescence from the dye was compared with reference upconversion signal to calibrate the performance of pH-sensor. The UC luminescence at 550 nm upon excitation with 980 nm light, was transferred to excite the indicator, emitting luminescence at 590 nm. 

**Figure 6:**
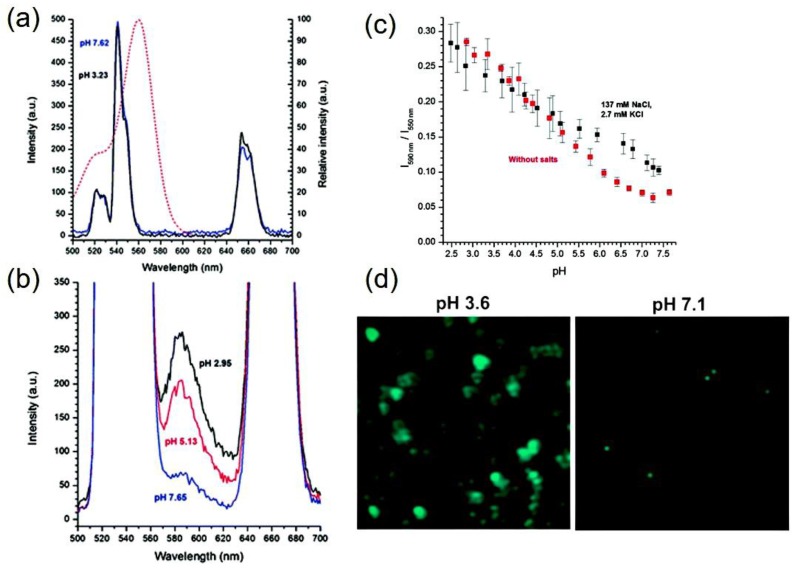
(**a**) Upconversion luminescence spectrum of dye conjugated-UCNPs in pH 7.62 and 3.23. The excitation spectrum of pHrodo™ Red (red dots, right *y*-axis) is overlapped with the upconversion emission spectrum. (**b**) Upconversion-sensitized luminescence emission spectrum of pHrodo™ Red in pH 7.65, 5.13 and 2.95. (**c**) Ratio of intensities of the sensitized luminescence emission of pHrodo™ Red at 590 nm and the upconversion emission at 550 nm with (black) and without (red) a physiological salt concentration. (**d**) Fluorescence microscopy images of the green luminescence of pHrodo™-conjugated-UCNPs at different pHs (Reproduced from Ref. [85] with permission from The Royal Society of Chemistry).

The pH-dependent luminescence spectra from pH 3.23 to pH 7.62 show that the dye emission increases in acidic condition (caused by protonation of the rhodamine chromophore), while no change is observed in the UC emission at 550 nm, as shown in Figure 6a,b. As only few upconversion emitters transfer their energy to the dye, most of the nanoparticles remain unaffected, giving non-reduction in 550 nm emission band. However, at higher concentration of the dye it is expected that attenuation of UCNPs’ emission at 550 nm will be prominent.

To identify the energy-transfer to dye further experiments were performed by time-resolved measurement, which shows that lifetime of the 550 nm emitting band in dye-conjugated UCNPs and only UCNPs have three components. However, one of these three components shortened to 58.6 µs from 94.0 µs in dye-conjugated UCNPs as compared to bare UCNPs, while the other two lifetime components remain unchanged (213 and 454 µs) in both types of UCNPs. The radiationless energy transfer has been considered as the reason behind the decrease in lifetime value by 35.4 µs [85].

In the further continuation of the study [85], the calibration curve shows linear response utilizing the ratio of dye emission (590 nm) to UC emission (550 nm) within pH 3 to 6.7, and nonlinear behavior in the rest region within pH 2.5 to 7.2. The pH-dependent confocal microscopy images of 550 nm emission of dye-conjugated UCNPs (concentration, 0.02 mg/mL) in saline shows that at low pH (pH 3.6) particle aggregation increases and decreases at high pH (pH 7.1) (Figure 6d). The experiment shows a linear change in the ratio of 590 nm to 550 nm emission, through analyzing of luminescence spectra from single aggregates.

Although this study seems to have good results, further work must be done to improve the dye emission through more effective energy transfer from the UCNPs. The aggregation of UCNPs in the system disturbs the evaluation measurement. Therefore, in a major advance in 2016 [31], the nanoparticles of NaYF_4_; Er^3+^/Yb^3+^-pHrodo^TM^ red were improved via surface modification by polyethyleneimine (PEI), covalently linked to the dye. Though PEI is cytotoxic in individual form, the toxicity is diminished when they are bound to the surface of UCNPs [31]. Surface modification through PEI has some other benefits, for instance, it increases cellular uptake by generating a large number of positive charges on the surface, improves the stability of the colloidal UCNPs, enhanced resonance energy transfer to the dye owing to strong covalent bonding by the reactive amino groups of PEI. Interestingly, the number of dye molecules was found to increase by 400 molecules/particle when the aminosilane was replaced by PEI, providing that the amount of dye is equal (67 nmol dye/1 mg of UCNP-PEI) in both the cases [31]. In addition, due to strong bonding of the dye with amine groups, a clear luminescence from the dye is observed in dye conjugated UCNP-PEI. The lifetime measurements show only a small change (14%) in one component of the decay time of 550 nm emitting level, while the other two components remain unaltered, as observed in the case of aminosilane coated UCNPs. Notwithstanding, a major reason for decreasing dye emission from aminosilane coated or PEI coated UCNPs has been concluded as the effect of quenching of luminescence by water molecules; since the resonance energy transfer is maximum at the surface of the nanoparticles [31]. Furthermore, the calibration curves in cell culture medium for aminosilane coated and PEI coated UCNPs display that the pH-sensitivity is greatly enhanced in the latter case, specifically in the neutral region of pH (pH 6.5–7) [31,85]. 

**Figure 7:**
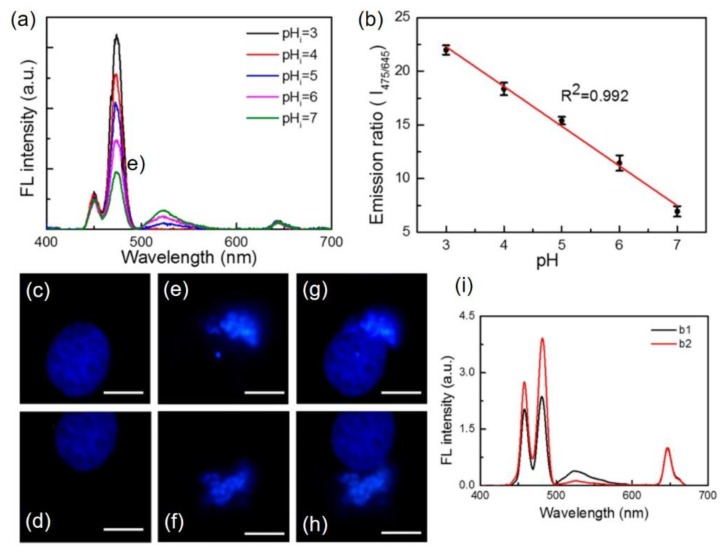
(**a**) Red emission normalized luminescence spectra of QBC939 cells (incubated with UCNPs) under 980 nm excitation at different pH. (**b**) Variation of the relative luminescence intensity ratio of 475 nm to 645 nm at different pH values: (**c**,**d**) images of the QBC939 cell’s nucleus under 405 nm excitation; (**e**,**f**) the upconversion images of different sites in single cell upon 980 nm light excitation; and (**g**,**h**) merged images of (**c**&**e**) and (**d**&**f**), respectively, in 10 µm scale bar. (**i**) The upconversion emission spectra of (**e**,**f**) sites of the cell, normalized at 645 nm (Reproduced from Ref. [30], Creative Commons license: http://creativecommons.org/licenses/by/4.0/).

Throughout our review, we have noticed that, in most of the studies, poor acceptor–donor pairs give low sensitivity due to the lack of proper overlap of the absorption and emission bands of indicator and UC emitter, respectively. The long separation between the acceptor and donor is concerned as to hinder the energy transfer. The Förster resonance energy transfer (FRET) was sufficiently increased in a PEI-modified NaYF_4_; Tm^3+^/Yb^3+^-fluorescein isothiocyanate pH-nanoprobe [30], showing a change of ratio by 3.56 unit per unit change in pH within 3.0 to 7.0 pH range in intracellular pH calibration measurements, as shown in Figure 7a,b. The nanoprobe is based on the change in UC luminescence at 475 nm (transfer energy to the indicator) and a reference signal at 650 nm, both originated from Tm^3+^ ions. Confocal microscopy images in the complex microenvironment of QBC939 cell, upon incubation with the designed nanoprobes show that the nanoparticles can detect bio-images at various sites of the living cells (Figure 7c–h) [30]. Collecting the blue band under 405 nm and 980 nm excitation, the images of the nucleus of the cell are shown in Figure 7c–f, respectively, along with their merged images in Figure 7g,h. The data from the images taken upon 980 nm light excitation were extracted as two spectra which exhibit pH-dependent behavior of the two sites of the nanoparticles and the calculated pH values according to calibration curves are 6.4 and 4.8 for e and f sites, respectively (Figure 7i). 

However, engineering the structure of UCNPs to achieve high UC emission and FRET efficiency is one of the most required criteria for figuring out efficient pH-nanoprobe. An effort to the light manipulation at the nanoscale is evident in the recent report by Du et al. [87]. The system is composed of NaGdF_4_:Tm^3+^/Yb^3+^@NaGdF_4_:Nd^3+^/Yb^3+^@NaYF_4_, coated with PEI (polyethyleneimine) and combined with fluorescein. The system is designed in such a way that it can be excited by both 808 and 980 nm light. The outer shell of NaYF_4_, protects the nanoparticle and manage the emitting ions away from surface defects and solvent. The Nd^3+^ ions are excited by 808 nm and sensitize the Yb^3+^ through Nd^3+^→Yb^3+^ energy transfer. While the Yb^3+^ ions are directly excited by 980 nm. The energy-transfer from Yb^3+^ to Tm^3+^ activators gives its characteristic emission in the blue region (450 and 475 nm) and red region (645 nm). The 450 nm and 475 nm luminescence bands are used to excite pH-sensitive dye via FRET, while the emission at 645 nm remains unaffected and serves as a reference signal for ratiometric determination of pH. In addition to the ratiometric pH-sensing, using the fluorescein emission at 515 nm and UC emission 645 nm, the system offers in vitro cell imaging with a confocal laser scanning microscope [30].

Another class of core@shell@shell structure of PAA-modified NaGdF_4_:Tm^3+^/Yb^3+^@NaYF_4_ conjugated with hemicyanine were prepared [88], where Tm^3+^/Yb^3+^ are in the shell so that these donor ions are adjacent to the acceptor dye molecules. The outer shell of NaYF_4_ serves as a support to intensify the total emission. The dye-conjugated UCNPs shows that the UC emission at 450 and 475 nm decreases in acidic medium, due to absorption of the dye at those wavelengths, while in neutral and basic medium the UC emissions at 475 and 513 nm decreases. At the same time, the emission band at 655 nm does not change, indicating that this wavelength is affected by the dye in neither acidic pH nor basic pH. This as-development pH-probe shows its reversibility in imaging of live-cell and is enabled to monitor pH-value through the variation of UC luminescence within pH 6.8–9. However, the problem that comes out from the distribution of the nanoparticles in living cell is that a small number of nanoparticles enter the living cells in acidic medium and confines their utilization in practical application [3] and it is expected that with the rapid progress in synthesis of biocompatible nanoparticle, this drawback can be resolved. 

**Figure 8:**
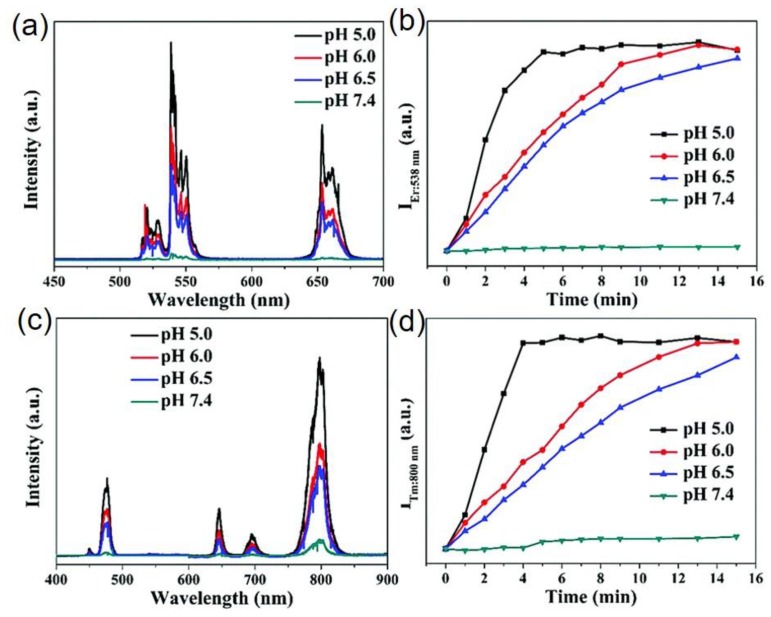
Upconversion response of: (**a**) NaYF_4_:Er^3+^/Yb^3+^@NaYF_4_@Ni; and (**c**) NaYF_4_:Tm^3+^/Yb^3+^@NaYF_4_@Ni nanoparticles in different pH solution. The plot of upconversion emission intensity a:t (**b**) 538 nm of Er^3+^; and (**d**) 800 nm of Tm^3+^ ions doped with Ni-modified UCNPs in different pH buffers versus of reaction time (Reproduced from Ref. [33] with permission from The Royal Society of Chemistry).

An alternative hybrid upconverting nanostructure consisting of NaYF_4_:Er^3+^/Yb^3+^@NaYF_4_@Ni was developed based on quenching and recovery of UC luminescence in basic and acidic solutions [33]. The hybrid nanoparticles (molar ratio, UCNPs/Ni = 1:0.28) in phosphate buffer solutions of pH 5.0, pH 6.0, pH 6.5 and pH 7.4 show that the upconversion is almost completely quenched at pH 7.4 (basic) and, as the buffer is changed towards pH 5.0 (acidic), the UC luminescence gets recovered gradually. The increase in UC luminescence intensity at the acidic medium is considered as the result of decomposition of Ni- nanoparticles and the UCNPs were gradually uncovered, resulting appearance of intrinsic UC luminescence. Figure 8 shows the spectra of the nanohybrids at different pH and the recovery times for the UC luminescence in different buffer solutions. It is worth noting that this is the second study on UC-based pH sensors without conjugating with pH-sensitive dye.

## 5. Biosafety of Upconversion Based Probes

The bio-safety of the UCNPs is the most important study for their practical application inside human body. Here, we will discuss the cytotoxicity, bio-distribution inside body and excretion of the UCNPs in the light of some conclusions of recent research. The toxicity and biodistribution of nanoparticles depend on several factors including composition of the nanoparticles, surface chemistry, size, and shape, along with the contaminants and their byproducts [89,90].

### 5.1. Toxicity Analysis of Upconversion Nanoparticles

**Figure 9:**
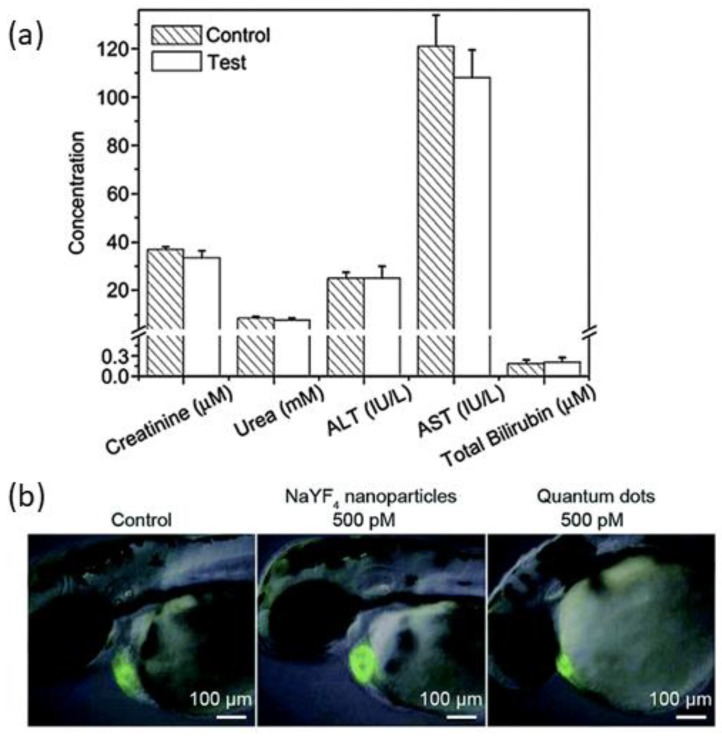
(**a**) Serum indicators of mice injected with PAA-conjugated NaYF_4_:Tm^3+^/Yb^3+^ UCNPs and mice receiving no injection (control); and (**b**) observation of heart function and heart size of zebrafish upon treatment with quantum dots and NaYF_4_ UCNPs (Figure 9a,b was reproduced from Ref. [94,97], Copyright (2010), (2014), respectively, with permission from Elsevier).

In standard assessing method for the analysis of toxicity, UCNPs with various concentrations are incubated with cells several times and the cell viability is measured to know their effect [56,91]. Cell viability for UCNPs is observed at their high concentration, which reflects their low cytotoxicity. The low cytotoxicity was measured for NaYF_4_:Tm^3+^/Yb^3+^@Fe_x_O_y_, which is less than that of iron oxide, a widely known non-toxic material demonstrated for human body application [92]. To get more accurate results, data are taken for a long time based on several evaluation parameters, such as hematological, histological and serum assays along with body weight (Figure 9a) [93]. The PAA-functionalized NaYF_4_:Tm^3+^/Yb^3+^ nanoparticles have shown that the weight of mice changes by a little amount without any other significant body effect, when injected intravenously and the mice lived for almost four months [94,95]. To detect the damage of tissue or inflammation, a histological evaluation was carried out on mice for the change in tissue of kidney, lung, heart, liver and spleen upon the exposure of PAA-functionalized NaYF_4_:Tm^3+^/Yb^3+^UCNPs and no damage of tissue was observed. In fact, there was no evidence of toxicity effect in mice when they were injected for seven days with 3 mg UCNPs daily [96].

In a separate study [97], hematological assay of PAA-conjugated NaYF_4_:Tm^3+^/Yb^3+^ UCNPs gives evidence that the shape and number of blood cells (RBC and WBC) and blood platelets remain unaltered, evaluating no noticeable toxicity in mice. For the determination of change in kidney and hepatic functions, studies were conducted on the relevant indicators for almost four months of exposure of UCNPs on mice gave no negative impact to the mice. Evaluation of heart function in zebrafish was performed using UCNPs (NaYF_4_:Tm^3+^/Yb^3+^) and compared with the treatment of quantum dots by Jang et al. [97], as shown in Figure 9b. The morphological effect of UCNPs and quantum dots were noticed after treatment of equal concentration (500 pM) of both the nanomaterials in separate tests. It shows (Figure 9b) that due to the quantum dots, the heart size is reduced, missing of embryos-looping and obstructed growth in the organ, while the UCNPs do not show any alteration in the morphology. This study further concludes that the effect of UCNPs is realized at very high concentration (~10 times) compared to quantum dots [98].

**Figure 10:**
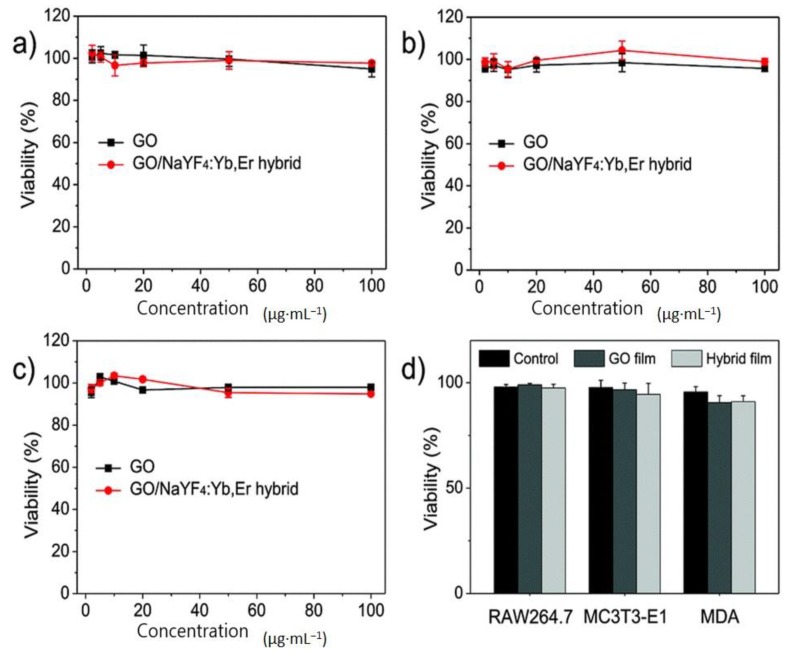
Biocompatibility of graphene oxide-PEI-NaYF_4_:Er^3+^/Yb^3+^ hybrid and hybrid film. (**a**–**c**) In vitro relative cellular viability of three types of cells (RAW264.7, MC3T3-E1 and MDA) treated with graphene oxide and the graphene oxide–NaYF_4_:Er^3+^/Yb^3+^ hybrid, respectively. (**d**) Cytotoxicity of pure graphene oxide and graphene oxide–NaYF_4_:Er^3+^/Yb^3+^ films (reproduced from Ref. [32] with permission from the PCCP Owner Societies).

Toxicity analysis of graphene oxide-NaYF_4_:Er^3+^/Yb^3+^-PEI hybrid UCNPs without containing any pH-indicator shows sufficient bio-compatibility [32]. The cell viability rests at 95% (even at 100 µg·mL^−1^) where the cell viability was at 100% initially (Figure 10). Three cell lines—RAW264.7, MDA and MC3T3-E1—were treated for 48 h with graphene oxide and hybrid upconverting film. It confirmed that the cells were grown on the film, leading to conclude that the hybrid upconverting nanomaterials are non-cytotoxic, where more than 95% of cells remain alive. 

The cytotoxicity of silica-coated Y_2_O_3_:Tm^3+^-conjugated with xylenol orange dye [79], evaluated for HeLa cells, after incubation with the UCNPs (concentration, 100 µg·mL^−1^) for one day, shows above 95% of the cells remained alive. Further incubating with concentrations up to 600 µg·mL^−1^ gives 80% cell viability for the same period. Presence of silica shell in these UCNPs is assumed to provide excellent biocompatibility. However, it is likely to decrease the energy transfer efficiency [79].

The confocal microscopy images of pHrodo^TM^ Red dye conjugated NaYF_4_:Er^3+^/Yb^3+^ hexagonal nanocrystals reveal that more than 90% of HeLa cells had internalized UCNPs [85]. Additionally, clustering of particles was observed due to endosomal compartmentalization. The size of cluster becomes bigger with the early endosome to late endosome fusion. This study shows absence of cytotoxicity even at concentration as high as 50 mg·mL^−1^ for a period of 16 h. The cellular uptake of NaYF_4_:Er^3+^/Yb^3+^ UCNPs were further improved with their coating with PEI instead of aminosilane, through the production of positive charge in PEI-UCNPs [31]. It is also found that the acidification time for endosomal compartments becomes slow using PEI through the proton sponge mechanism.

### 5.2. Bio-Distribution and Excretion of Upconversion Nanoparticles

Intravenous and intra-arterial are two types of injection pathways for nanoparticles inside body that are commonly followed in general studies [56]. The UCNPs in different organs show different trend of bio-distribution. The surface properties of the UCNPs are modified for their in vivo applications. For example, PEG (polyethylene glycol) is a common surface ligand that is considered to extend the circulation time of the nanoparticles. Jalil et al. [99] investigated the post-injection distribution of silica modified UCNPs (NaYF_4_:Er^3+^/Yb^3+^@SiO_2_) in lung, kidney, heart, spleen, liver and blood. The amount of yttrium, as determined was 29.2, 18.0 mg·L^−1^·g^−1^ after 10 min of injection in lung and heart, respectively. After 24 h, these amounts reduced to 0.45 and 0.04 mg·L^−1^·g^−1^ in those organs, respectively. The kidney displayed no change in UCNPs concentration (17.7 mg·L^−1^·g^−1^) at 24 h of injection, while the nanoparticles concentration in spleen was 6.3 mg·L^−1^·g^−1^ after 30 min of injection. However, low concentration of UCNPs appeared in blood and liver during the experiment. The distributions of UCNPs in organs have been observed as non-static and it fluctuates with time as proven in several works [100]. 

The distribution of PEI-coated NaYF_4_:Er^3+^/Yb^3+^ UCNPs, calculated according to their pH value as measured through ratiometric way shows that in nigericin treated cells, the percentage of total population increases for cytoplasmic, endosomal and lysosomal compartments of cell compared to untreated cells (Table 3) [31].

**Figure 11:**
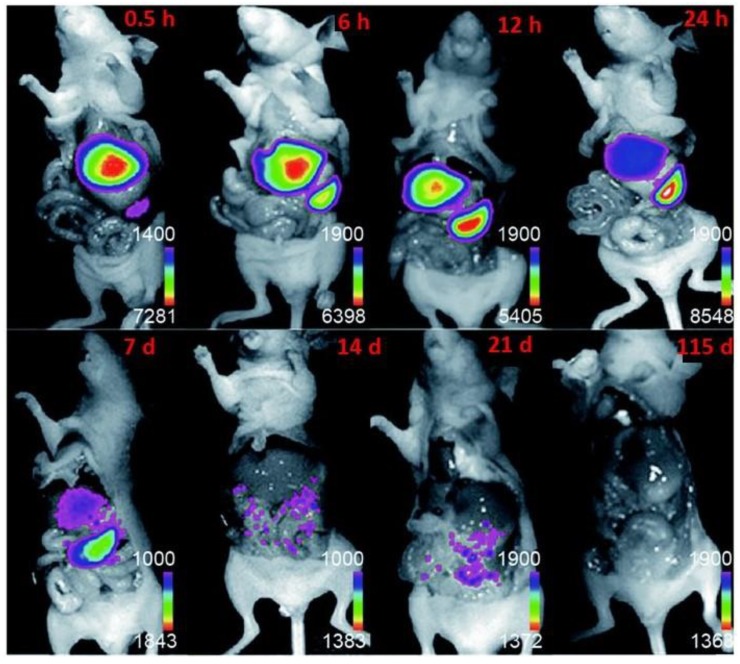
Upconversion imaging of mice with injection of PAA-coated NaYF_4_:Tm^3+^/Yb^3+^ UCNPs at various time points (Reproduced from Ref. [94], Copyright (2010), with permission from Elsevier).

The nanoparticles injected into body should be removed completely within a certain time. Most drugs are cleared from body by biliary or renal excretion [101]. Foreign nanoparticles are removed by phagocytosis process. In biliary excretion, the nanoparticles are catabolized through hepatocytes. The nanoparticles, which are not affected by intercellular process, remain in the body for a long time [94]. The distribution of UCNPs and their removal was imaged in mice using NaYF_4_:Tm^3+^/Yb^3+^-PAA UCNPs (15 mg·kg^−1^), injected through the tail vein. After seven days of injection, the transport of UCNPs was noticed to the intestinal tract and after 21 days, the UCNPs only remained in the intestinal tract. After persisting at the same part for 90 days, the UCNPs entirely cleared from the body of mice after 115 days (Figure 11). At the same time, some other investigations show that the shape and size of the PEG-modified UCNPs remain unaffected after excreted through biliary route [56,102]. Another convenient passage for UCNPs removal is renal excretion, giving rise to the opportunity of reduction in cytotoxicity, though it depends on the size of the particles to be excreted. The level of toxicity depends on the time of UCNPs remain in the body before its eradication [103]. For large particles, it takes several weeks to months to years to be removed via biliary route, while the UCNPs via renal route take only a few hours to days to be excreted [94]. 

The excretion time of lung is faster than spleen and liver. For example, in spleen and liver, the decrease of NaY(Gd)F_4_:Tm^3+^/Yb^3+^ UCNPs are 43% and 33%, respectively, while 57% decrease of the nanoparticles were observed in the lung [104,105]. Currently, the fastest removal time for UCNPs is reported as seven days for PEI-modified NaYF_4_:Er^3+^/Yb^3+^@SiO_2_ nanoparticles with diameter 50 nm [106].

## 6. Conclusions

Recently, several reviews have focused on the synthesis and application (other than pH-sensing) of upconversion materials [25,26,27,28]. In this review, we present the recent progress in upconversion sensitized pH-nanoprobes, along with their effect in living cell. The groundwork in the field of intercellular pH-probe was done by the group of Kopelman [107,108] using a pH-sensitive fluorophore together with a reference one. Due to the absence of autofluorescence, photobleaching and blinking nature, UCNPs are a favorable class of nanoparticles that promises to have significant impact in sensing and therapy in living cells. Although extensive studies are being carried out from inter-disciplinary field of researchers, the research in this field is still open to be developed for their practical applications. To design a better nanoprobe for pH-sensing for living cell, several inter-cellular requirements should be fulfilled, e.g., good colloidal stability, high cellular uptake, strong brightness, photostability and non-cytotoxicity [31]. The UCNPs-sensitized pH-sensors are of special interest because other inorganic pH-sensors, such as quantum dots or carbon nanodots, are lacking advantages that UCNPs have [23,24]. The common problem appearing to researchers while internalizing the nanoprobes inside cell is that, due to internalization through endocytosis process, nanoparticles are not homogenously distributed inside, rather they remain in specific parts of the cells (e.g., in acidic vesicles), which are unwanted sites for the experimental goal and could prevent determining the endosomal compartments correctly [3]. Control and monitor of exact pH in different cellular components is necessary for living being. Cell proliferation and movements can also be organized by monitoring pH value. Cellular dysfunction generally occurs due to uncontrolled variation of pH. Therefore, internalization of the nanoprobes in cells should be further studied and developed to fulfill a successive conclusion for the application of nanoparticles [30].

The problem confronted by the researchers in upconversion based intracellular, accurate pH-detection is that the calibration of pH in buffer is not always realizable due to deviation, originated from the continuous generation of protons and their absorption into charged UCNPs (e.g., in PEI-coated UCNP), combined with buffer mediated pH-variation. Although quantitative pH control is known inadequately, the protonation of endosomes and lysosomes is steady state due to proton pumping V-ATPase (ATPase), and pH measurement encounters with overstated pH determination [31,109]. In conjunction with this, the measuring instruments exhibit individual and inconsistent amplification of optical signals. In the future, the targeting of pH-nanoprobes into specific tissues is the next challenge in constructing pH-nanoprobes. The in vivo variation of pH in ischaemia or cancerous tumor tissues is essential in biomedical therapy [3]. Especially in medical research, in vivo imaging of pH of the target gives details of the tissues. The pH-nanoprobes should provide high contrast for in vivo imaging in tumor cells. The nanoprobes can also be combined with drug delivery where acidic pH generally causes the release of drug. In the case of core-shell nanoparticles, the bio-functionalization should be done properly so that these can be applied to specific tissues. Notwithstanding, the pH-nanoprobes should be highly photostable for in vivo imaging [23].

The quantification of intensity ratio requires a complete understanding of the limitations and components of the involved system. The errors in pH-sensing may be introduced by several factors, e.g., the sample, the detection system, experimental environment, laser excitation density, etc. The errors in pH-measurement may reflect the error in the pH-value (inaccuracy) and/or imprecision (variation in the pH-value upon repeated measurement). Thus, the accuracy and precision are both equally meaningful here. The ratiometric method eliminates the interference caused due to the external and environmental influences such as fluctuation of excited light. In addition, for the quantitative determination of intracellular pH, the calibration experiments should be done extensively. In the case of single-wavelength based pH-sensors, the influence of the non-pH dependent factors such as concentration of nanoparticles, excitation power, temperature of the vicinity, etc. induces error in the pH-measurement. The pH-determination according to calibration curve carried out in buffer may not provide the actual pH in intra-cellular compartment because of the difference in proton production as well as absorption of protons to the UCNPs’ surface coating and pH-change mediated by buffers [3]. 

Most of the UC-based pH-sensors are constructed on the FRET process. The capability of providing FRET efficiency is another criterion because UCNPs are used as donor to the pH-dependent indicator in FRET process. Generally, the energy transfer process between UCNPs and indicator is limited within few nanometer (1–10 nm) [40,41,42,43]. On the other hand, the luminescence intensity depends on the nanoprobes as well. Therefore, the concentration of the compositional elements, FRET efficiency, and dimension of the nanoparticles must be homogeneous in nature. Furthermore, some other points should be given attention for the design of intracellular pH-sensing: (i) the excitation and emission signals should penetrate the biological tissue with minimized auto-fluorescence; (ii) the sensors should take into account the total emission intensity because of attenuation of signals in tissue; and (iii) the spectral distribution and error in calibration due to absorption and scattering of light by tissue must be minimized. 

Although the design of the pH-nanoprobes and complexity of the specimens have impacts on the resolution and accuracy of pH-sensing, as an improvement to obtain higher accuracy, the intensity ratio could be plotted into a probability density function with Gaussian distribution fitting [31]. However, due to proton-sponge effect a slight variation may experience in the calibration curve [31]. The developed sensors must be stable, selective, accurate, reproducible and should have reasonable response time. Attenuation of light takes place through some absorption and scattering events by the biological tissues [110]. In addition, the lower irradiance than the threshold value of the excitation wavelength is expected to be attenuated in the tissue. To reduce the laser induced heating effect, core-shell structures should be preferred for designing the nanoparticles. In intracellular pH-measurement, the biological cells also have natural viability along with the variance caused by measurement techniques. Therefore, to attain highly accurate and precise value of pH, these sources of inaccuracy and imprecision must be reduced in the measurements. 

Therefore, in the current perspective, new biocompatible functional nanomaterials must be engineered with the advances of nanotechnology to obtain highly accurate novel pH nanoprobes for their excellent performance at the intercellular and extracellular level in biomedical research. A challenging issue in this field is to focus the pH-sensors based on UC without use of dye and the next few years is likely to witness considerable progress in this field.

## Figures and Tables

**Table 1:** Emission bands of the rare earth doped some host materials.

Host Material	Dopant Ions	Major Emission Bands			References
		Blue	Green	Red	
β-NaYF_4_	Yb^3+^/Er^3+^		525, 545	655	[61]
β-NaYF_4_	Yb^3+^/Tm^3+^	461, 478	527	650	[62]
β-NaYF_4_	Tm^3+^/Eu^3+^	450, 475			[63]
NaGdF_4_	Ho^3+^/Yb^3+^	486	541	647, 751	[64]
NaYbF_4_	Yb^3+^/Ho^3+^	480	541	645	[65]
YF_3_	Pr^3+^	483, 485			[66]
LaF_3_	Yb^3+^/Tm^3+^	475.2	698	800.2	[67]
LaF_3_	Yb^3+^/Ho^3+^		542.4	644.7, 657.8	[68]
LaF_3_	Yb^3+^/Er^3+^		520.8, 545	658.8	[69]

**Table 2:** Upconversion based pH-sensors with their response time, range and method.

pH-Probe		Response Time	pH Range	Method	References
UCNPs	Indicator				
NaYF_4_:Er^3+^/Yb^3+^	bromothymol blue	N/A	2–11	Absolute intensity of 548 nm and 658 nm bands	[78]
Y_2_O_2_S:Er^3+^/Yb^3+^ (film)	Bromocresol green	~10 min	5–10	Ratio of 671 nm to 661 nm	[34]
NaYF_4_:Er^3+^/Yb^3+^ with graphene oxide (film)	(No dye)	1 min	5–8	Changes in emission intensity of 540 nm	[32]
NaYF_4_:Er^3+^/Yb^3+^ aminosilane coated	pHrodo^TM^ Red	N/A	2.5–7.2	Ratio of UC emission at 540 nm to the dye emission at 590 nm	[85]
NaYF_4_:Er^3+^/Yb^3+^-PEI	pHrodo^TM^ Red	N/A	5–7	Ratio of UC emission at 540 nm to the dye emission at 590 nm	[31]
NaYF_4_:Tm^3+^/Yb^3+^	Fluoroscein isothiocynate	N/A	3–7	Ratio of UC emission at 475 nm to 645 nm	[30]
Y_2_O_3_Tm^3+^@SiO_2_	Xylenol orange	N/A	4–8	Ratio of UC emission at 450 nm and 646 nm	[79]
NaGd(WO_4_)_2_:Eu^3+^	Fluoroscein isothiocyanate allylamine hydrocloride	N/A	4–10	Ratio of fluoroscein emission at 512 nm to RE emission at 611 nm	[80]
NaYF_4_:Er^3+^/Yb^3+^	Bromothymol blue	N/A	6–8	Absolute UC emission at 650 nm	[81]
Commercial phosphor:Er^3+^ (film)	Neutral red	23 s	5–8.5	Ratio of green to red UC emission	[83]
NaYF_4_:Er^3+^/Yb^3+^@NaYF_4_@Ni	(No dye)	N/A	5–7.4	Quenching and recovery of green and red UC bands	[33]
NaGdF_4_@NaYF_4_:Yb^3+^/Tm^3+^@NaYF_4_-PAA	hemicyanine		6.8–9	Ratio of 650 nm to 513 nm	[88]
NaYF_4_:Er^3+^/Yb^3+^(film)	ETH 5418	N/A	6–11	Ratio of UC emission bands at 656 nm to 542 nm	[84]
NaGdF_4_:Tm^3+^/Yb^3+^@NaGdF_4_:Nd^3+^/Yb^3+^ @NaYF_4_-PEI	Fluorescein	N/A	4.7–7.4	Ratio of dye emission at 515 nm to UC emission at 645 nm	[87]

**Table 3:** Ratio of luminescence intensity of 590 nm to 550 nm in different cellular pH ranges before and after treatment with 20 μM nigericin (adapted with permission from Ref. [31]. Copyright (2017) American Chemical Society).

pH	Intensity Ratio	Untreated Cells		Nigericin Treated Cells	
		No. of Events	% of Total Population	No. of Events	% of Total Population
7.2–7.5	<0.1	41	5	12	9
6.0–7.2	0.1–0.4	637	77	119	91
<6.0	>0.4	144	17	0	0
